# The effects of incomplete protein interaction data on structural and evolutionary inferences

**DOI:** 10.1186/1741-7007-4-39

**Published:** 2006-11-03

**Authors:** Eric de Silva, Thomas Thorne, Piers Ingram, Ino Agrafioti, Jonathan Swire, Carsten Wiuf, Michael PH Stumpf

**Affiliations:** 1 Theoretical Genomics Group, Centre for Bioinformatics, Division of Molecular Biosciences, Imperial College London, London, UK; 2 Department of Mathematics, Imperial College London, London, UK; 3 Bioinformatics Research Center, University of Aarhus, Aarhus, Denmark; 4 Molecular Diagnostic Laboratory, Aarhus University Hospital, Aarhus, Denmark; 5 Institute of Mathematical Sciences, Imperial College London, London, UK

## Abstract

**Background:**

Present protein interaction network data sets include only interactions among subsets of the proteins in an organism. Previously this has been ignored, but in principle any global network analysis that only looks at partial data may be biased. Here we demonstrate the need to consider network sampling properties explicitly and from the outset in any analysis.

**Results:**

Here we study how properties of the yeast protein interaction network are affected by random and non-random sampling schemes using a range of different network statistics. Effects are shown to be independent of the inherent noise in protein interaction data. The effects of the incomplete nature of network data become very noticeable, especially for so-called network motifs. We also consider the effect of incomplete network data on functional and evolutionary inferences.

**Conclusion:**

Crucially, when only small, partial network data sets are considered, bias is virtually inevitable. Given the scope of effects considered here, previous analyses may have to be carefully reassessed: ignoring the fact that present network data are incomplete will severely affect our ability to understand biological systems.

## Background

Molecular networks such as protein interaction, transcriptional or metabolic networks are widely seen as integrative and coherent descriptions for the whole complement of molecular processes inside a cell [1]. There has been considerable interest in their structure, their functional organization and their evolutionary properties. For important model organisms such as *Saccharomyces cerevisiae, Caenorhabditis elegans *and *Drosophila melanogaster *there are now extensive protein interaction data deposited in public-domain databases and serious attempts are being made at elucidating the human protein interaction network (PIN) [2,3]. These network data sets – extensive though they are thanks to experimental advances and *in silico *prediction – do not cover the entire network. In particular they do not include all the proteins in these organisms and represent samples only from much larger networks.

But a network introduces a set of relationships and potential dependencies between the constituent nodes and these may be broken up in the subnet. By subnet we mean a subset S
 MathType@MTEF@5@5@+=feaafiart1ev1aaatCvAUfKttLearuWrP9MDH5MBPbIqV92AaeXatLxBI9gBamrtHrhAL1wy0L2yHvtyaeHbnfgDOvwBHrxAJfwnaebbnrfifHhDYfgasaacH8akY=wiFfYdH8Gipec8Eeeu0xXdbba9frFj0=OqFfea0dXdd9vqai=hGuQ8kuc9pgc9s8qqaq=dirpe0xb9q8qiLsFr0=vr0=vr0dc8meaabaqaciaacaGaaeqabaWaaeGaeaaakeaaimaacqWFse=uaaa@3845@ of the nodes of the overall global network N
 MathType@MTEF@5@5@+=feaafiart1ev1aaatCvAUfKttLearuWrP9MDH5MBPbIqV92AaeXatLxBI9gBamrtHrhAL1wy0L2yHvtyaeHbnfgDOvwBHrxAJfwnaebbnrfifHhDYfgasaacH8akY=wiFfYdH8Gipec8Eeeu0xXdbba9frFj0=OqFfea0dXdd9vqai=hGuQ8kuc9pgc9s8qqaq=dirpe0xb9q8qiLsFr0=vr0=vr0dc8meaabaqaciaacaGaaeqabaWaaeGaeaaakeaaimaacqWFneVtaaa@383B@ and the interactions among them *(i.e*. the induced subgraph of a set of nodes); depending on how the nodes in S
 MathType@MTEF@5@5@+=feaafiart1ev1aaatCvAUfKttLearuWrP9MDH5MBPbIqV92AaeXatLxBI9gBamrtHrhAL1wy0L2yHvtyaeHbnfgDOvwBHrxAJfwnaebbnrfifHhDYfgasaacH8akY=wiFfYdH8Gipec8Eeeu0xXdbba9frFj0=OqFfea0dXdd9vqai=hGuQ8kuc9pgc9s8qqaq=dirpe0xb9q8qiLsFr0=vr0=vr0dc8meaabaqaciaacaGaaeqabaWaaeGaeaaakeaaimaacqWFse=uaaa@3845@ are chosen, properties of S
 MathType@MTEF@5@5@+=feaafiart1ev1aaatCvAUfKttLearuWrP9MDH5MBPbIqV92AaeXatLxBI9gBamrtHrhAL1wy0L2yHvtyaeHbnfgDOvwBHrxAJfwnaebbnrfifHhDYfgasaacH8akY=wiFfYdH8Gipec8Eeeu0xXdbba9frFj0=OqFfea0dXdd9vqai=hGuQ8kuc9pgc9s8qqaq=dirpe0xb9q8qiLsFr0=vr0=vr0dc8meaabaqaciaacaGaaeqabaWaaeGaeaaakeaaimaacqWFse=uaaa@3845@ will be different from those of N
 MathType@MTEF@5@5@+=feaafiart1ev1aaatCvAUfKttLearuWrP9MDH5MBPbIqV92AaeXatLxBI9gBamrtHrhAL1wy0L2yHvtyaeHbnfgDOvwBHrxAJfwnaebbnrfifHhDYfgasaacH8akY=wiFfYdH8Gipec8Eeeu0xXdbba9frFj0=OqFfea0dXdd9vqai=hGuQ8kuc9pgc9s8qqaq=dirpe0xb9q8qiLsFr0=vr0=vr0dc8meaabaqaciaacaGaaeqabaWaaeGaeaaakeaaimaacqWFneVtaaa@383B@. Until very recently, all studies surprisingly ignored the effects of the incompleteness of molecular networks [4] despite the fact that the sampling properties of networks can lead to systematic differences between the properties of networks and their subnets (discrepancies can be further inflated when the nodes in S
 MathType@MTEF@5@5@+=feaafiart1ev1aaatCvAUfKttLearuWrP9MDH5MBPbIqV92AaeXatLxBI9gBamrtHrhAL1wy0L2yHvtyaeHbnfgDOvwBHrxAJfwnaebbnrfifHhDYfgasaacH8akY=wiFfYdH8Gipec8Eeeu0xXdbba9frFj0=OqFfea0dXdd9vqai=hGuQ8kuc9pgc9s8qqaq=dirpe0xb9q8qiLsFr0=vr0=vr0dc8meaabaqaciaacaGaaeqabaWaaeGaeaaakeaaimaacqWFse=uaaa@3845@ are chosen in a highly ascertained manner [5]). While random subnets of classical random graphs have properties that can be taken as representative of the true network, most networks, notably the popular scale-free classes of networks, will display noticeable and qualitative differences between networks and their subnets. This early work was followed by an analysis of Han *et al*. [6], who reported results regarding the effects of sampling on the degree distribution of PINs and further theoretical studies by Lee *et al*. [7]; Hakes *et al*. [8] considered not subsampling but the question of the effect of data-set selection on structural inferences of networks, which can also have considerable impact on the analysis and may explain differences between analyses. A host of other network statistics can be considered in addition to the degree distribution, Pr(*k*), in order to assess the structure [9]; these include the clustering coefficient and network motifs (see *Methods *for definitions). Importantly, all of these will be different for subnets compared with the true network and it is essential to understand the extent to which subnet properties other than the degree distribution differ from those of the true network. As we will show, this is to a large extent a question of how the subnet is created (that is, how nodes are chosen), and the statistic under consideration. A useful general premise we have found is that subnetworks differ more from the true network in non-local properties: *i.e*. their degree distributions will be more "similar" (in a loose sense which has been made somewhat more precise [5,10]) than, for example, motif spectra [11,12].

It is thus important to understand the extent to which the sampling properties of networks affect our inferences regarding structure, function and evolution. Considerable effort has been invested in understanding, for example, the functional organization and evolutionary properties of PINs, and contradictory results have been reported in the literature which are probably affected by many factors in addition to incomplete data. We have recently studied statistical sampling properties of network ensembles [4,5] in considerable detail: the results suggest that when ≳80% of the nodes in a network are sampled at random, the shape of the degree distribution of the subnet, Pr*(*k*), will be virtually indistinguishable from that of the true network. Current PIN data comprise interactions only among a relatively small number of the proteins known to be present in the different organisms. For *S. cerevisiae*, for which sampling is most complete, present publicly available data sets include interaction data among ≈4900 out of an estimated 6000 proteins. We have therefore taken the present *S. cerevisiae *PIN as a starting point for our analysis. We compare results for subnets with those of the assumed 'true' network. This study is meant as a qualitative investigation into how incomplete sampling has affected studies into PINs and not as a quantitative assessment of the reliability of the present dataset. Despite the noise in the present yeast PIN, the *S. cerevisiae *data will give us a more realistic representation of a true PIN than theoretical network models.

We will show that the sampling nature of a real network does indeed lead to different properties in the subnets compared with the true network. Sampling properties of networks have hitherto been largely ignored – whereas the poor data quality has attracted considerable attention [13,14] – but may lead to large variances and biases for network statistics obtained for different subnets, and act independently of noise. In light of the present analysis it may be necessary to reevaluate previous results for biological networks. In the context of systems biology this study demonstrates both the importance of performing carefully delimited studies of well-defined aspects of systems, and the potential pitfalls of analyzing only parts or components of complex biological systems. Clearly the way the data have been collected needs to be considered before an analysis, and the sampling properties of networks need to be included in the analysis explicitly and from the outset.

## Results

### Network sampling schemes

Assuming random sampling of nodes leads to great simplifications in the mathematical analysis [4]. In reality, however, experimenters are more likely to pick some proteins than others and quite generally we can assume that each protein has probability 0 <*p*_*i *_< 1. Then the number of nodes in the subnet is given by

NS=∑i=1Npi     (1)
 MathType@MTEF@5@5@+=feaafiart1ev1aaatCvAUfKttLearuWrP9MDH5MBPbIqV92AaeXatLxBI9gBamrtHrhAL1wy0L2yHvtyaeHbnfgDOvwBHrxAJfwnaebbnrfifHhDYfgasaacH8akY=wiFfYdH8Gipec8Eeeu0xXdbba9frFj0=OqFfea0dXdd9vqai=hGuQ8kuc9pgc9s8qqaq=dirpe0xb9q8qiLsFr0=vr0=vr0dc8meaabaqaciaacaGaaeqabaWaaeGaeaaakeaacqWGobGtdaWgaaWcbaacdaGae8NeXpfabeaakiabg2da9maaqahabaGaemiCaa3aaSbaaSqaaiabdMgaPbqabaaabaGaemyAaKMaeyypa0JaeGymaedabaGaemOta4eaniabggHiLdGccaWLjaGaaCzcamaabmaabaGaeGymaedacaGLOaGaayzkaaaaaa@480A@

Equally, we can determine the average probability of sampling a node

p¯=1N∑i=1Npi.     (2)
 MathType@MTEF@5@5@+=feaafiart1ev1aaatCvAUfKttLearuWrP9MDH5MBPbIqV92AaeXatLxBI9gBaebbnrfifHhDYfgasaacH8akY=wiFfYdH8Gipec8Eeeu0xXdbba9frFj0=OqFfea0dXdd9vqai=hGuQ8kuc9pgc9s8qqaq=dirpe0xb9q8qiLsFr0=vr0=vr0dc8meaabaqaciaacaGaaeqabaqabeGadaaakeaacuWGWbaCgaqeaiabg2da9maalaaabaGaeGymaedabaGaemOta4eaamaaqahabaGaemiCaa3aaSbaaSqaaiabdMgaPbqabaaabaGaemyAaKMaeyypa0JaeGymaedabaGaemOta4eaniabggHiLdGccqGGUaGlcaWLjaGaaCzcamaabmaabaGaeGOmaidacaGLOaGaayzkaaaaaa@3FA2@

As *N *becomes large (strictly as *N *→ ∞) it is possible to show that we can use p¯
 MathType@MTEF@5@5@+=feaafiart1ev1aaatCvAUfKttLearuWrP9MDH5MBPbIqV92AaeXatLxBI9gBaebbnrfifHhDYfgasaacH8akY=wiFfYdH8Gipec8Eeeu0xXdbba9frFj0=OqFfea0dXdd9vqai=hGuQ8kuc9pgc9s8qqaq=dirpe0xb9q8qiLsFr0=vr0=vr0dc8meaabaqaciaacaGaaeqabaqabeGadaaakeaacuWGWbaCgaqeaaaa@2E2D@ rather than the individual *p*_*i *_to determine the sampling probabilities of random networks.

#### Sampling properties of networks

Uncorrelated random networks are networks which are maximally random conditional on a given degree distribution [15,16] (thus their degree-degree correlations may be different from zero); in such a case it is possible to express expectation values of many interesting network characteristics in terms of the degree distribution Pr(*k*); more interestingly, the degree sequence is a sufficient (see *e.g*. Cox and Hinkley [17]) statistic for uncorrelated networks. We can straightforwardly calculate the first two moments of the degree distribution in the subnet [5], Pr*(*k*):

〈k〉S=p〈k〉N     (3)
 MathType@MTEF@5@5@+=feaafiart1ev1aaatCvAUfKttLearuWrP9MDH5MBPbIqV92AaeXatLxBI9gBamrtHrhAL1wy0L2yHvtyaeHbnfgDOvwBHrxAJfwnaebbnrfifHhDYfgasaacH8akY=wiFfYdH8Gipec8Eeeu0xXdbba9frFj0=OqFfea0dXdd9vqai=hGuQ8kuc9pgc9s8qqaq=dirpe0xb9q8qiLsFr0=vr0=vr0dc8meaabaqaciaacaGaaeqabaWaaeGaeaaakeaacqGHPms4cqWGRbWAcqGHQms8daWgaaWcbaacdaGae8NeXpfabeaakiabg2da9iabdchaWjabgMYiHlabdUgaRjabgQYiXpaaBaaaleaacqWFneVtaeqaaOGaaCzcaiaaxMaadaqadaqaaiabiodaZaGaayjkaiaawMcaaaaa@4A76@

〈k2〉S=p2〈k2〉N+p(1−p)〈k〉N,     (4)
 MathType@MTEF@5@5@+=feaafiart1ev1aaatCvAUfKttLearuWrP9MDH5MBPbIqV92AaeXatLxBI9gBamrtHrhAL1wy0L2yHvtyaeHbnfgDOvwBHrxAJfwnaebbnrfifHhDYfgasaacH8akY=wiFfYdH8Gipec8Eeeu0xXdbba9frFj0=OqFfea0dXdd9vqai=hGuQ8kuc9pgc9s8qqaq=dirpe0xb9q8qiLsFr0=vr0=vr0dc8meaabaqaciaacaGaaeqabaWaaeGaeaaakeaacqGHPms4cqWGRbWAdaahaaWcbeqaaiabikdaYaaakiabgQYiXpaaBaaaleaaimaacqWFse=uaeqaaOGaeyypa0JaemiCaa3aaWbaaSqabeaacqaIYaGmaaGccqGHPms4cqWGRbWAdaahaaWcbeqaaiabikdaYaaakiabgQYiXpaaBaaaleaacqWFneVtaeqaaOGaey4kaSIaemiCaaNaeiikaGIaeGymaeJaeyOeI0IaemiCaaNaeiykaKIaeyykJeUaem4AaSMaeyOkJe=aaSbaaSqaaiab=1q8obqabaGccqGGSaalcaWLjaGaaCzcamaabmaabaGaeGinaqdacaGLOaGaayzkaaaaaa@5CFF@

where <...> denotes the sample mean and *p *the sampling probability. These equations are true whether a network is uncorrelated or not.

As the sampling fraction increases from zero to one the sampeld network will undergo a structural phase transition [18,19] in the limit *N *→ ∞. One of the main consequences is the emergence of the giant connected component [18]. This is present (for *N *→ ∞) when the average number of next-nearest neighbours, *z*_2 _of a random node is on average greater than the number of its nearest neighbours *z*_1_; *i.e*.

*z*_2 _> *z*_1 _    (5)

The number of nearest and next-nearest neighbours in a network N
 MathType@MTEF@5@5@+=feaafiart1ev1aaatCvAUfKttLearuWrP9MDH5MBPbIqV92AaeXatLxBI9gBamrtHrhAL1wy0L2yHvtyaeHbnfgDOvwBHrxAJfwnaebbnrfifHhDYfgasaacH8akY=wiFfYdH8Gipec8Eeeu0xXdbba9frFj0=OqFfea0dXdd9vqai=hGuQ8kuc9pgc9s8qqaq=dirpe0xb9q8qiLsFr0=vr0=vr0dc8meaabaqaciaacaGaaeqabaWaaeGaeaaakeaaimaacqWFneVtaaa@383B@ are given by

*z*_1 _≡ <*k*>     (6)

and

*Z*_2 _≡ <*k*^2^> - <*k*>,     (7)

respectively. Substituting Eqns. (3) and (4) yields for condition (5) in the subnet

p>〈k〉N〈k2〉N−〈k〉N=z1,Nz2,N.     (8)
 MathType@MTEF@5@5@+=feaafiart1ev1aaatCvAUfKttLearuWrP9MDH5MBPbIqV92AaeXatLxBI9gBamrtHrhAL1wy0L2yHvtyaeHbnfgDOvwBHrxAJfwnaebbnrfifHhDYfgasaacH8akY=wiFfYdH8Gipec8Eeeu0xXdbba9frFj0=OqFfea0dXdd9vqai=hGuQ8kuc9pgc9s8qqaq=dirpe0xb9q8qiLsFr0=vr0=vr0dc8meaabaqaciaacaGaaeqabaWaaeGaeaaakeaacqWGWbaCcqGH+aGpdaWcaaqaaiabgMYiHlabdUgaRjabgQYiXpaaBaaaleaaimaacqWFneVtaeqaaaGcbaGaeyykJeUaem4AaS2aaWbaaSqabeaacqaIYaGmaaGccqGHQms8daWgaaWcbaGae8xdX7eabeaakiabgkHiTiabgMYiHlabdUgaRjabgQYiXpaaBaaaleaacqWFneVtaeqaaaaakiabg2da9maalaaabaGaemOEaO3aaSbaaSqaaiabigdaXiabcYcaSiab=1q8obqabaaakeaacqWG6bGEdaWgaaWcbaGaeGOmaiJaeiilaWIae8xdX7eabeaaaaGccqGGUaGlcaWLjaGaaCzcamaabmaabaGaeGioaGdacaGLOaGaayzkaaaaaa@602B@

Thus the sampling fraction *p *for which the subnet does not have a GCC depends in an intuitive and simple manner on the properties of the overall network N
 MathType@MTEF@5@5@+=feaafiart1ev1aaatCvAUfKttLearuWrP9MDH5MBPbIqV92AaeXatLxBI9gBamrtHrhAL1wy0L2yHvtyaeHbnfgDOvwBHrxAJfwnaebbnrfifHhDYfgasaacH8akY=wiFfYdH8Gipec8Eeeu0xXdbba9frFj0=OqFfea0dXdd9vqai=hGuQ8kuc9pgc9s8qqaq=dirpe0xb9q8qiLsFr0=vr0=vr0dc8meaabaqaciaacaGaaeqabaWaaeGaeaaakeaaimaacqWFneVtaaa@383B@. For the yeast PIN considered here the GCC will cease to exist for *p *≲ 0.041. For classical or Erdös-Rényi random graphs, where the degree distribution is given by a Poisson distribution with parameter *λ *(for large N) equation (8) means that the sampling fraction must exceed *p *> 1λ
 MathType@MTEF@5@5@+=feaafiart1ev1aaatCvAUfKttLearuWrP9MDH5MBPbIqV92AaeXatLxBI9gBaebbnrfifHhDYfgasaacH8akY=wiFfYdH8Gipec8Eeeu0xXdbba9frFj0=OqFfea0dXdd9vqai=hGuQ8kuc9pgc9s8qqaq=dirpe0xb9q8qiLsFr0=vr0=vr0dc8meaabaqaciaacaGaaeqabaqabeGadaaakeaadaWcaaqaaiabigdaXaqaaGGaciab=T7aSbaaaaa@2F67@ for a GCC to exist.

### Subnet structures

A random subnet comprising *e.g*. *p *= 60% of the nodes of the true network differs quite substantially from the true network (here *p *is the probability of sampling a node; the fraction of nodes included in the subnet is binomially distributed with probability *p*). The graph induced by the subset of nodes has a substantially smaller number of edges than the sampling fraction, *p *(see Table 1). For example, for *p *= 60% slightly more than a third of the interactions will be observed. Trying to predict the size of interactomes by linear extrapolation from present data sets will thus underestimate the true interactome size [20]. For random sampling, however, it is in fact straightforward to predict the number of interactions: if a fraction *p *of nodes has been sampled, then the fraction of edges that has been sampled is simply the fraction of pairs of nodes, *i.e*. a random subnet with sampling probability *p *will have a proportion of *p*^2 ^of the edges. For *S. cerevisiae *we have thus 15,181 out of approximately 15,181/0.80^2 ^≈ 23,800 interactions (which are detectable given current experimental technology).

#### Degree distribution

**Figure 1:**
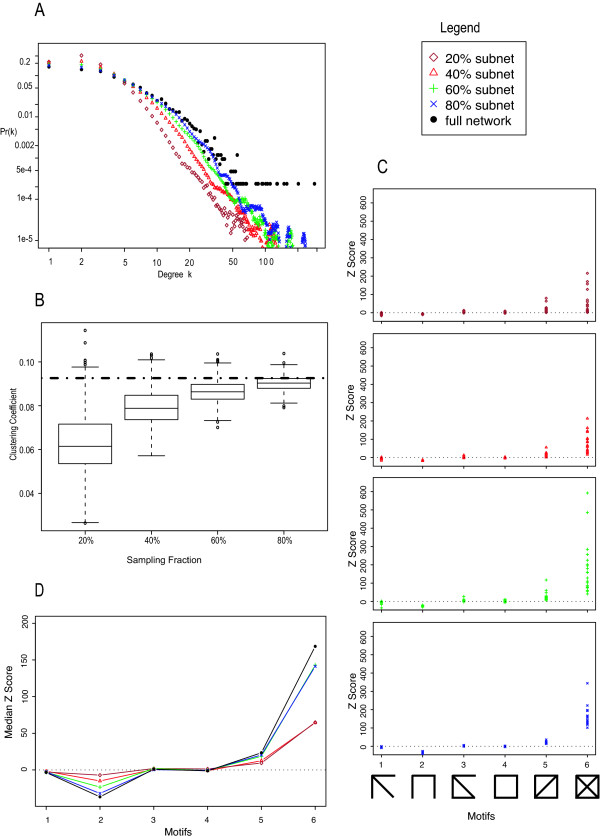
**Properties of the yeast protein interaction networks under random sampling**. (A) The degree distribution for the full network and the average for the subnets (averaged over the ensemble) generated by sampling 80%, 60%, 40% and 20% of the nodes in the *Saccharomyces cerevisiae *protein interaction network. Nodes with degree *k *= 0 have been dropped from the analysis, reflecting the content of interaction databases. (B) The horizontal line shows the clustering coefficient of the full network. From the boxplots it is apparent that with decreasing subnet size the clustering coefficient will tend to decrease, reflecting the increasingly sparse network with a correlated structure. (C) *Z*-scores for the six 4-motifs in the true network and 20 random subnets for sampling fractions *p *= 80%, 60%, 40% and 20%. (D) Median *Z*-scores for each motif in each of the subnet ensembles and the *Z*-score of the motif in the full network. In (C) and (D) a positive *Z*-score indicates that the motif is overrepresented in the true network compared with randomly rewired versions of the true network; a negative *Z*-score indicates under-representation.

In Figure 1A, as the sampling fraction decreases statistical weight tends to flow from high degrees to low degrees (we have removed nodes with *k *= 0 from the degree distribution). Moreover, at low degrees the degree distribution appears to become more power law-like as the sampling fraction decreases; this is a curious point given claims about scale-free properties of so many biological networks that are effectively subnets of the real network. Previous analyses [4,5] show, however, that even the degree distributions of subnets are generally qualitatively different from those of the true network; in particular if the degree distribution of the network takes on a power law form, the subnet (as the value of *p *decreases) will have a qualitatively different degree distribution and *vice versa*.

**Figure 2:**
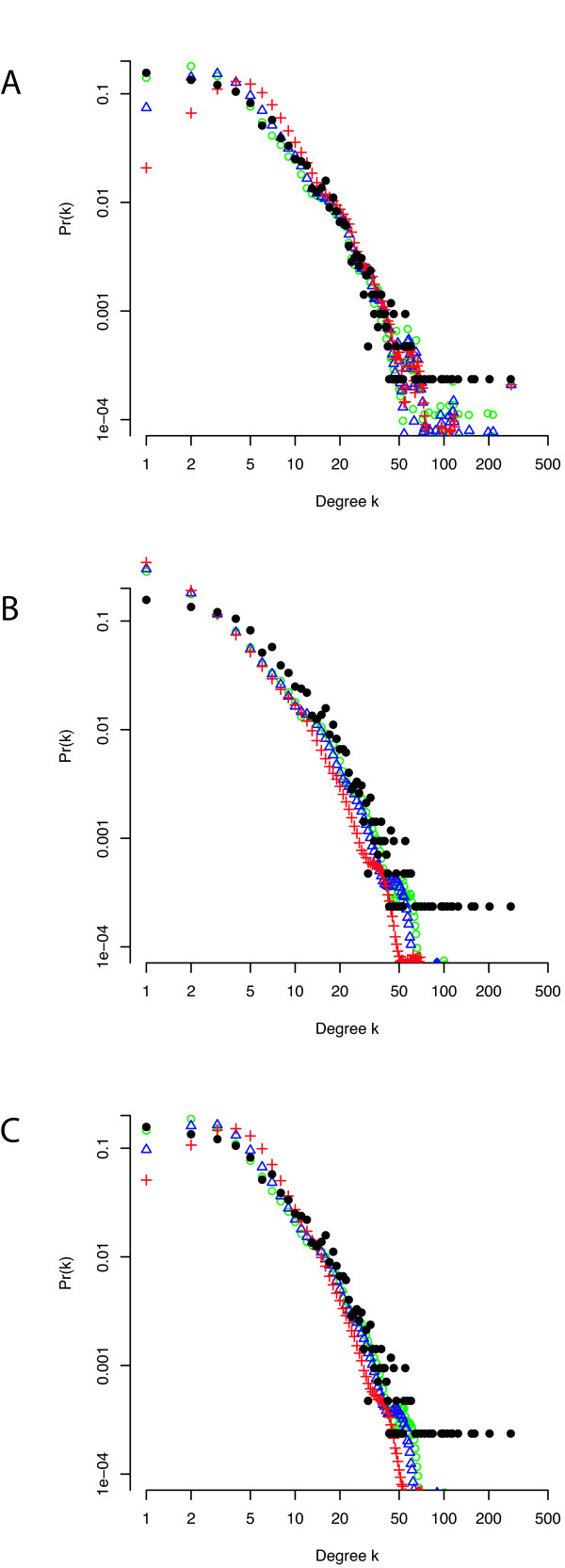
**Degree distribution of noisy yeast protein interaction networks**. Degree distributions for "noisy" networks with 10% (green), 20% (blue) and 40% (red) false-positives (A), false negatives (B) and rewired edges (C). In each case the degree distribution of the true network is shown in black. Shown are averages obtained from 1000 independent instances. The 95% CIs of the degree distributions overlap the symbols used to indicate the mean, *i.e*. the variance of Pr(*k*) at degree *k *is relatively small.

On average a node with degree *k *in the global network will have degree *pk *[4,5] in a randomly sampled subnet (with sampling fraction *p*) and the peaks that are visible in the tail of the subnet degree distributions correspond to the most highly connected nodes in the full network: the maximum degree is 283 and corresponding peaks appear at ≈226, ≈170, ≈113 and at ≈57, for sampling fractions of 80%, 60%, 40% and 20%, respectively, that were generated by random selection of nodes with probability *p*. Because of the binomial sampling procedure used in generating networks, (where the degree distribution in the subnet Pr⁡S(k)
 MathType@MTEF@5@5@+=feaafiart1ev1aaatCvAUfKttLearuWrP9MDH5MBPbIqV92AaeXatLxBI9gBamrtHrhAL1wy0L2yHvtyaeHbnfgDOvwBHrxAJfwnaebbnrfifHhDYfgasaacH8akY=wiFfYdH8Gipec8Eeeu0xXdbba9frFj0=OqFfea0dXdd9vqai=hGuQ8kuc9pgc9s8qqaq=dirpe0xb9q8qiLsFr0=vr0=vr0dc8meaabaqaciaacaGaaeqabaWaaeGaeaaakeaacyGGqbaucqGGYbGCdaWgaaWcbaacdaGae8NeXpfabeaakiabcIcaOiabdUgaRjabcMcaPaaa@3E22@ is given by Eqn. (10) (see *Methods*), the most highly connected nodes will remain the same – as will their rank order and the relative proportion – in the subnets as in the global network, provided, of course, that they are included in the subnet; see Figure 2.

The effects of noise on the network data are shown in Figure 2 where we have added, subtracted and rewired, respectively, a fraction of the interactions among nodes. Qualitatively, we find that the shoulder of the degree distribution (*i.e*. the shape of the distribution at intermediate values of the degree *k*) is only little affected. Particularly at low, but also at high degree, the shape of the distribution may also differ quite considerably. Thus noise should generally distort the degree distribution in a different way from the way incomplete network data do.

#### Clustering coefficients

Figure 1B shows the spread of the average clustering coefficient in the four subnet ensembles. The horizontal line shows the empirical clustering coefficient of the full network. In the supplementary material [see Additional file 1] we show that for large uncorrelated uniform networks [21] the clustering coefficient does not change at all under random sampling. The systematic decrease in the average clustering coefficients with decreasing subnet size reflects the presence of degree-degree correlations (previously shown by Agrafioti *et al*. [22]) in the network data. We also also observe an increase in spread and range with decreasing subnet size. The empirical clustering coefficient (indicated by the horizontal line) is higher than the median, but falls within the distribution of clustering coefficient (*C*) values obtained for all subnet ensembles, suggesting that correlations in the network are not very strong. This is in contrast to Figure 1A, where the degree distribution is more globally affected. This and the behaviour of *C *under sampling in the giant connected component are discussed further in the supplementary material.

#### Betweenness

The dependence of betweenness or betweenness-centrality (BC; see *Methods*) on the sampling fraction is more subtle than that of the degree or clustering coefficient as it also depends on the global structure of the network. Thus, for example, in different subnet samples the 10 proteins with the highest BC values change much more than the 10 proteins with the highest degrees. However, a very good correlation (Kendall's *τ *≳ 0.79 in the true network) between degree and BC is seen for all values of *p *(data not shown).

#### Motifs

In this study we pay particular attention to the six motifs defined by four nodes in an undirected graph (illustrated at the bottom of Figure 1C). The observed range of the *Z*-scores (see *Methods*) for all motifs considered here decreases with subnet size. For each subnet size we observe considerable spread in the range of *Z*-scores for the different motifs shown in Figure 1C. Motifs 1,3 and 4 can have both negative and positive *Z*-scores depending on the sample (motif 4 has positive and negative statistically significant *Z*-scores even for 80% subnets in the 20 subnets studied here). For motif 6, the most highly connected, we observe the biggest spread as well as a general increase in the average *Z*-score with subnet size. In Figure 1D we observe that the median *Z*-score for motif 6 is the same in both the 20% and 40% subnets, and the 60% and 80% subnets, respectively. This is, however, entirely due to chance and to the high variance of motif *Z*-scores in random subnets as is shown by further analyses (see supplementary material [See Additional file 1]). The importance of network data integrity and completeness is further exemplified by comparing the results in figure 1C with those in the original papers by Milo *et al*. [11,12]; here effects of the choice of data set also come into play [8].

### Non-random ascertainment schemes

**Figure 3:**
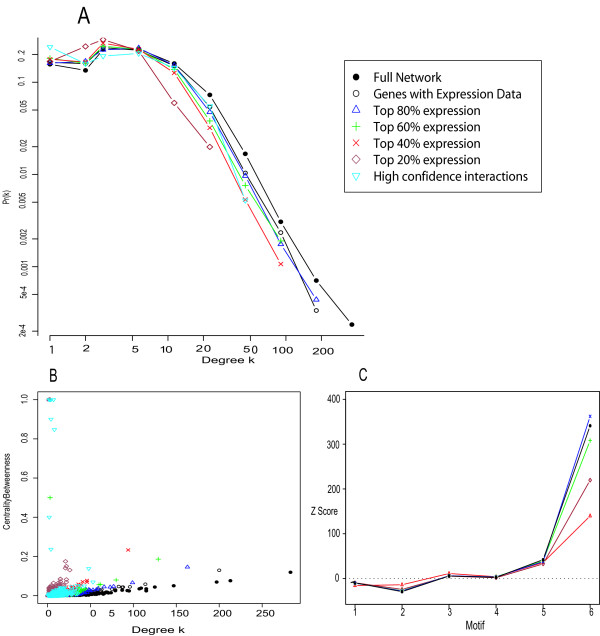
**Properties of the yeast protein interaction networks under non-random sampling**. (A) Degree distributions for proteins with different expression levels and a subnet generated from interactions which have previously been assigned as more reliable. (B) Betweenness-centrality for the same subnets. (C) Z-score of each of six different 4-motifs for the full network and each subnet sampled according to expression level, as well as the network consisting of high-confidence interaction data.

The degree distributions differ quite considerably between the different sampling schemes (see Figure 3A). It is particularly interesting to note that the high-confidence data network has the degree distribution which is most similar to the degree distribution of the complete data-set. BC is shown in part B of the same figure which confirms the results outlined above: there is a systematic increase with decreasing sampling fraction *p *or subnet size. There are some nodes which appear to be on the shortest paths between all (or almost all) pairs of nodes. These do not, however, correspond to the most highly connected nodes, but rather occur for low degrees (*k *= 2).

For the subnets constructed on the basis of protein expression data, we determined the 4-motif *Z*-scores. In Figure 3C it can be seen that all the motifs have similar *Z*-scores in the different data sets except for the fully connected 4-motif. The *Z*-scores of this motif do not exhibit a simple ordering, *e.g*. the subnet comprising the 80% of nodes with the highest expression levels exhibits higher *Z*-scores than the subnet consisting of all nodes where expression level data is available. Finally, this network has a *Z*-score for motif 6 that is twice as high as that obtained for the full network. We also detect some systematic differences for motifs 1, 3 and 4. These had *Z*-scores ≈ 0 in the true network and all randomly generated subnets (Figure 3C), but have negative *Z*-scores in the networks which are based on expression level. This suggests that experimental bias in designing interactome mapping studies will lead to systematic differences in motif spectra for different sampling schemes.

### Incomplete data and functional and evolutionary inferences

So far, we have considered only structural properties of networks. The interest in molecular networks lies, however, in the hope that they can explain the mechanisms underlying complex biological processes. Their impact on the evolutionary properties of molecules has also been studied and here we seek to understand how informative inferences from subnets are about the properties of larger networks.

**Figure 4:**
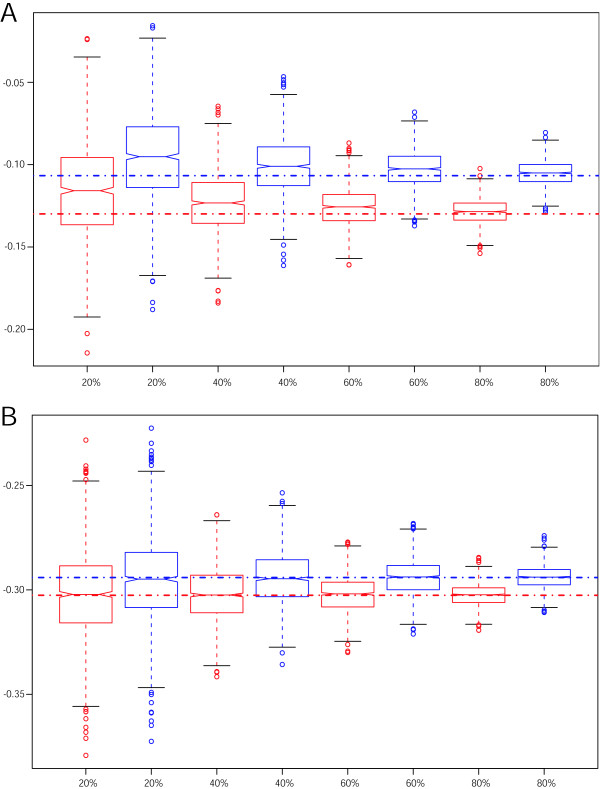
**Correlation between evolutionary rate and degree and expression level**. (A) The boxplots of Kendall's rank correlation coefficient (red) show a weak anti-correlation between evolutionary rate and degree, which increases with subnet size. The corresponding partial correlation coefficients (blue) indicate a weaker anti-correlation when protein expression level is controlled for. (B) Correlation coefficients (red) between evolutionary rate and protein expression level and partial correlation coefficients (blue) which account for differences in protein degree. The anti-correlations found here are stronger than those shown in part A of this figure. The horizontal dot-dashed lines represent the correlation coefficients of the full network.

Figure 4A shows the correlation and partial correlation (correcting for expression level variation) coefficients between evolutionary rate and degree for the 20%, 40%, 60% and 80% subnetworks; correlations and partial correlations are measured using Kendall's rank correlation coefficient, *τ*. The evolutionary rate is obtained from comparisons with six other yeast species [22], based on reconstructed phylogenies. There is a weak anti-correlation between evolutionary rate and degree and this anti-correlation is further weakened when expression level is taken into account in the partial correlation coefficients (blue boxplots in Figure 4). This anti-correlation strengthens somewhat in the larger subnets. There is a stronger anti-correlation (see Figure 4B) between evolutionary rate and expression level. These results suggest that the qualitative results of the work of, for example, Agrafioti *et al*. [22] – at least those referring to single nodes – remain valid in the ensembles of random subnets. Quite generally, under random sampling of nodes, single-node properties or any qualities that depend on a protein's degree should also be observable in the subnet. For example, under random sampling the most common proteins will remain the same, provided, of course, that they are included in the subnet (Table 2). Because of random sampling, a node which has rank *m *in the list of nodes ordered by degree in the full network will have rank *l *<* m *in a subnet with probability

πm,l=(ml−1)pl−1(1−p)m−l+1     (9)
 MathType@MTEF@5@5@+=feaafiart1ev1aaatCvAUfKttLearuWrP9MDH5MBPbIqV92AaeXatLxBI9gBaebbnrfifHhDYfgasaacH8akY=wiFfYdH8Gipec8Eeeu0xXdbba9frFj0=OqFfea0dXdd9vqai=hGuQ8kuc9pgc9s8qqaq=dirpe0xb9q8qiLsFr0=vr0=vr0dc8meaabaqaciaacaGaaeqabaqabeGadaaakeaaiiGacqWFapaCdaWgaaWcbaGaemyBa0MaeiilaWIaemiBaWgabeaakiabg2da9maabmaabaqbaeqabiqaaaqaaiabd2gaTbqaaiabdYgaSjabgkHiTiabigdaXaaaaiaawIcacaGLPaaacqWGWbaCdaahaaWcbeqaaiabdYgaSjabgkHiTiabigdaXaaakiabcIcaOiabigdaXiabgkHiTiabdchaWjabcMcaPmaaCaaaleqabaGaemyBa0MaeyOeI0IaemiBaWMaey4kaSIaeGymaedaaOGaaCzcaiaaxMaadaqadaqaaiabiMda5aGaayjkaiaawMcaaaaa@4CE4@

conditional on it being included in the subnet. Eqn. (9) reflects the obvious point that the average rank of a node decreases with decreasing sampling fraction *p*. But because of Eqn. (9), single node properties in the true network – *e.g*. frequency of protein domains [23] or correlation between degree and expression level [22] – will be statistically conserved in the subnets. We note that these results are qualitatively unaffected by the reported "stickiness" of some of the proteins in table 2 (Sticky proteins will, of course also be sticky in smaller yeast two-hybrid studies). In the table we also provide the number of interactions observed in the high-confidence Database of Interacting Proteins (DIP) [24] data set. We find that the number of interactions reported for these proteins decreases dramatically (more quickly than would be expected given the relative size of these datasets) but that overall we find a reasonable level correlation between the degrees of proteins which are included in both data sets (Kendall's *τ *≈ 0.53; *p *< 10^-10^). Discovering potential relationships between, for example, motifs and evolutionary and functional properties, as previously suggested [25], is subject to the more disruptive effects of network sampling on such structures (several studies have found other reasons why the functional interpretation of motifs may be difficult in many instances, see, for example, [26,27]). Given the results shown for motifs (discussed above in relation to Figures 1C,D and 2C), such analyses may need to be carefully re-evaluated in light of the sampling nature of present network data.

## Discussion

We have explored effects of sampling on statistical measures of protein interaction structure for different sampling schemes. Our comparison with the effects of noisy interaction data (see figure 2) suggests that sampling and noise affect network statistics in different ways and we have therefore concentrated on the sampling effects as noise has received considerable attention previously (see, for example, [28,13,29]). Previous studies of network sampling properties focused on the degree distribution [4,30,6]. In our analysis we confirmed the results of these earlier studies, but one aspect of this study deserves closer scrutiny: with decreasing sampling fraction the degree distribution of the randomly sampled subnets becomes straighter and the slope of the best-fit line becomes steeper. More interestingly, we find that for a data set which had previously [28] been classified as consisting of more reliable interactions, the degree distribution appears to be reasonably similar to the degree distribution of the overall network (this can be also quantified statistically [5]), especially when compared with the randomly generated subnetwork ensemble.

Not surprisingly, we find that the effects of sampling on other statistical measures such as clustering coefficient, betweenness and motifs are more intricate (average pathlengths and diameter [1] have similarly diverse sampling properties). As statistical measures become less local, the effects of sampling become increasingly subtle. For example, BC is a non-local property and the effects of sampling act locally as well as globally as the system undergoes a structural phase transition with the giant connected component [19,31] breaking up as *p *decrease. Thus the fraction of pairs of nodes which are connected (belong to the same component) decreases and an increasing fraction of nodes has a BC value of 0. On the other hand, the fraction of shortest paths passing through the connected nodes increases systematically.

Motifs are local objects [11,12,32] but *Z*-scores are constructed using a global network-rewiring approach [33,34]. Therefore their sampling properties are more intricate than those of subgraphs that are defined differently [35]. This dual nature of motifs – they are local objects but their significance is assessed against a globally randomized network ensemble – explains the qualitative differences in their behaviour under different sampling regimes.

In addition to the sampling properties, one result which becomes obvious from the present analysis is that subnets of the same size can differ quite considerably; and, in particular, the more complex measures of network structure such as motif spectra can exhibit variances that overwhelm the mean or median statistics. This becomes particularly apparent in Figure 1C. It is partially for this reason that we have not emphasised the non-random sampling schemes more: a single instance of a network statistic represents only an instance of a sample drawn from an ensemble; for networks sampling of nodes leads to very broad distributions of sample statistics as would be expected for such highly correlated and structured data sets [1]. Sampling and noise affect these network statistics differently, with incomplete data introducing variability as well as systematic bias, and noise affecting almost exclusively the variance in, for example, the *Z*-scores of motifs.

For random subnets we also compared evolutionary results previously obtained for the "complete network" for the randomly generated networks. In Agrafioti *et al*. [22] only the effects of local structure (*i.e*. degree) were used and in light of the previous discussion it is therefore not surprising that the central results are generally confirmed in the subnets: in particular protein expression level correlates better than degree with protein evolutionary/substitution rate. For the non-random sampling schemes the data are biased in favour of protein abundance and results are also confirmed, but potentially biased somewhat against degree. In general, single-node properties of proteins are statistically conserved in the subnet, *e.g*. the protein with the highest degree will, provided it is being included in the sample, tend to have the highest degree also in the subnet. As far as biological and functional inferences are concerned, the effects of network sampling properties appear to be not very different from statistical missing data problems. Thus the biological studies, which investigate, for example, the interplay between protein domain structure and protein interactions [23] are probably not affected. Investigating such properties across a network [36], however, may be subject to bias because of the intricacies displayed by the network sampling behaviour discussed here.

## Conclusion

In summary, our analysis shows that it is important to include the sampling nature of biological networks explicitly and from the outset. Failure to do so may have given rise to biases in previous network analyses. In particular this is the case for statistics which involve more than one node such as motif spectra [12] or pairwise similarities of nodes [37]. In other branches of the quantitative biosciences, notably population genetics [38], the effects of sampling and their importance are well understood. The same is not true for the fledgling field of systems biology.

Noise and incompleteness affect network data in subtly different ways. As we have shown here, a subnet is much less than a part of the whole network and failure to account for this will bias inferences.

## Methods

### Yeast protein interaction data

Protein-protein interactions of *Saccharomyces cerevisiae *are obtained from the DIP database which lists 4773 proteins ('nodes' in network parlance) and the 15,461 interactions observed between these proteins. It is a manually curated catalogue of protein complexes and the interactions are obtained, *inter alia*, from yeast two-hybrid experiments and literature extraction. It is estimated that *S. cerevisiae *has around 6000 genes, so that which we call the full network is really a subnetwork itself. We have removed self-interactions leaving 15,181 interacting protein pairs; self-interactions are removed so that we can describe the PIN in terms of a simple graph [18]. It should be noted that in PINs the rates for false-positive and false-negative results are estimated [13,39] to be around 40%, with many interactions endorsed by only one experimental observation. This dataset then constitutes our assumed "real" or complete network.

### Generating subnets

We randomly sampled (without replacement) the real network to produce 1000 subnets comprising 20%, 40%, 60% and 80% of the total number of nodes, respectively (Table 1). The random sampling scheme is the most parsimonious model for the choice of nodes which make up the subnets. In reality, however, experimentalists designing *e.g*. yeast two-hybrid experiments will be guided by prior knowledge and/or a particular biological question in mind. While it is difficult to model the precise ascertainment process we have some additional information which allows us to study the effects of two other ascertainment schemes: first we consider the networks generated by taking all proteins which were included in the expression analysis of Cho *et al*. [40] as well as the 20%, 40%, 60% and 80% of proteins with the highest expression levels. The second ascertainment scheme we consider is the subnet of protein interactions which have been deemed to be reliable in the analysis of Gavin *et al*. [28] (referred to in the main text as high-quality/high-confidence data).

### Generating noisy networks

The present *S. cerevisiae *PIN is, of course, not free from false-positive interactions; equally, false-negatives will have lead to missing interactions. Here we have used the PIN data as if it were the true network to study the effects of incomplete network data under different sampling schemes discussed above. In order to study the effects of noise, we follow the approach of Yook *et al*. [29] and add 10%, 20% and 40% of false interactions to study the effects of false-positives; we delete 10%, 20% and 40% of interactions to model the effect of false-negative; and we rewire (which corresponds to adding and deleting equal proportions of interactions) 10%, 20% and 40% of interactions to study the joint effects of false-positive and false-negative interactions. In this way we can qualitatively compare the effects of noise in the data with those of incomplete network data on network statistics.

### Degree distribution

The degree distribution, Pr(*k*), is the probability that a node has *k *interaction partners. In uncorrelated networks [41,16], other properties depend only on the degree distribution and the degree sequence is a sufficient statistic. The expected degree distribution in the subnet is given by

Pr⁡S(k)=∑l≥k(lk)pk(1−p)l−kPr⁡S(l),     (10)
 MathType@MTEF@5@5@+=feaafiart1ev1aaatCvAUfKttLearuWrP9MDH5MBPbIqV92AaeXatLxBI9gBamrtHrhAL1wy0L2yHvtyaeHbnfgDOvwBHrxAJfwnaebbnrfifHhDYfgasaacH8akY=wiFfYdH8Gipec8Eeeu0xXdbba9frFj0=OqFfea0dXdd9vqai=hGuQ8kuc9pgc9s8qqaq=dirpe0xb9q8qiLsFr0=vr0=vr0dc8meaabaqaciaacaGaaeqabaWaaeGaeaaakeaacyGGqbaucqGGYbGCdaWgaaWcbaacdaGae8NeXpfabeaakiabcIcaOiabdUgaRjabcMcaPiabg2da9maaqafabaWaaeWaaeaafaqabeGabaaabaGaemiBaWgabaGaem4AaSgaaaGaayjkaiaawMcaaaWcbaGaemiBaWMaeyyzImRaem4AaSgabeqdcqGHris5aOGaemiCaa3aaWbaaSqabeaacqWGRbWAaaGccqGGOaakcqaIXaqmcqGHsislcqWGWbaCcqGGPaqkdaahaaWcbeqaaiabdYgaSjabgkHiTiabdUgaRbaakiGbccfaqjabckhaYnaaBaaaleaacqWFse=uaeqaaOGaeiikaGIaemiBaWMaeiykaKIaeiilaWIaaCzcaiaaxMaadaqadaqaaiabigdaXiabicdaWaGaayjkaiaawMcaaaaa@6351@

or by

Pr⁡S(k)=11−∑l=0∞(1−p)lPr⁡N(l)∑l≥k(lk)pk(1−p)l−kPr⁡N(l),     (11)
 MathType@MTEF@5@5@+=feaafiart1ev1aaatCvAUfKttLearuWrP9MDH5MBPbIqV92AaeXatLxBI9gBamrtHrhAL1wy0L2yHvtyaeHbnfgDOvwBHrxAJfwnaebbnrfifHhDYfgasaacH8akY=wiFfYdH8Gipec8Eeeu0xXdbba9frFj0=OqFfea0dXdd9vqai=hGuQ8kuc9pgc9s8qqaq=dirpe0xb9q8qiLsFr0=vr0=vr0dc8meaabaqaciaacaGaaeqabaWaaeGaeaaakeaacyGGqbaucqGGYbGCdaWgaaWcbaacdaGae8NeXpfabeaakiabcIcaOiabdUgaRjabcMcaPiabg2da9maalaaabaGaeGymaedabaGaeGymaeJaeyOeI0YaaabmaeaacqGGOaakcqaIXaqmcqGHsislcqWGWbaCcqGGPaqkdaahaaWcbeqaaiabdYgaSbaakiGbccfaqjabckhaYnaaBaaaleaacqWFneVtaeqaaOGaeiikaGIaemiBaWMaeiykaKcaleaacqWGSbaBcqGH9aqpcqaIWaamaeaacqGHEisPa0GaeyyeIuoaaaGcdaaeqbqaamaabmaabaqbaeqabiqaaaqaaiabdYgaSbqaaiabdUgaRbaaaiaawIcacaGLPaaaaSqaaiabdYgaSjabgwMiZkabdUgaRbqab0GaeyyeIuoakiabdchaWnaaCaaaleqabaGaem4AaSgaaOGaeiikaGIaeGymaeJaeyOeI0IaemiCaaNaeiykaKYaaWbaaSqabeaacqWGSbaBcqGHsislcqWGRbWAaaGccyGGqbaucqGGYbGCdaWgaaWcbaGae8xdX7eabeaakiabcIcaOiabdYgaSjabcMcaPiabcYcaSiaaxMaacaWLjaWaaeWaaeaacqaIXaqmcqaIXaqmaiaawIcacaGLPaaaaaa@7B38@

if nodes of degree 0 in the subnet are ignored.

### Clustering coefficient

The clustering coefficient *C *is a measure of the average local neighbourhood in a network [42]. It is defined as the probability that two nodes *j *and *k *which are connected to node *i *are themselves connected to each other, and its value is restricted to the unit interval, 0 ≤ *C *≤ 1. It is averaged over all nodes in the network:

C=∑i=1N2×Number of neighbours of node i which are themselves neighbourski(k1−1)     (12)
 MathType@MTEF@5@5@+=feaafiart1ev1aaatCvAUfKttLearuWrP9MDH5MBPbIqV92AaeXatLxBI9gBaebbnrfifHhDYfgasaacH8akY=wiFfYdH8Gipec8Eeeu0xXdbba9frFj0=OqFfea0dXdd9vqai=hGuQ8kuc9pgc9s8qqaq=dirpe0xb9q8qiLsFr0=vr0=vr0dc8meaabaqaciaacaGaaeqabaqabeGadaaakeaacqWGdbWqcqGH9aqpdaaeWbqaamaalaaabaGaeGOmaiJaey41aqRaeeOta4KaeeyDauNaeeyBa0MaeeOyaiMaeeyzauMaeeOCaiNaeeiiaaIaee4Ba8MaeeOzayMaeeiiaaIaeeOBa4MaeeyzauMaeeyAaKMaee4zaCMaeeiAaGMaeeOyaiMaee4Ba8MaeeyDauNaeeOCaiNaee4CamNaeeiiaaIaee4Ba8MaeeOzayMaeeiiaaIaeeOBa4Maee4Ba8MaeeizaqMaeeyzauMaeeiiaaIaemyAaKMaeeiiaaIaee4DaCNaeeiAaGMaeeyAaKMaee4yamMaeeiAaGMaeeiiaaIaeeyyaeMaeeOCaiNaeeyzauMaeeiiaaIaeeiDaqNaeeiAaGMaeeyzauMaeeyBa0Maee4CamNaeeyzauMaeeiBaWMaeeODayNaeeyzauMaee4CamNaeeiiaaIaeeOBa4MaeeyzauMaeeyAaKMaee4zaCMaeeiAaGMaeeOyaiMaee4Ba8MaeeyDauNaeeOCaiNaee4CamhabaGaem4AaS2aaSbaaSqaaiabdMgaPbqabaGccqGGOaakcqWGRbWAdaWgaaWcbaGaeGymaedabeaakiabgkHiTiabigdaXiabcMcaPaaaaSqaaiabdMgaPjabg2da9iabigdaXaqaaiabd6eaobqdcqGHris5aOGaaCzcaiaaxMaadaqadaqaaiabigdaXiabikdaYaGaayjkaiaawMcaaaaa@9575@

where *k*_*i *_is the degree of node *i*. It is a measure which describes the average local structure in a network [1]. When *C *is calculated only for the giant connected component the behaviour will differ slightly (Supplementary material [See Additional file 1]).

### Betweenness

The betweenness of a node is the number of shortest paths in a network which includes this node [43]. Betweenness-centrality (BC) is the fraction of shortest paths which runs through a node. Here we focus on BC and its change under sampling. BC is highly correlated with degree in an obvious way with hubs having higher centrality than lower-degree nodes.

### Motifs

Motifs are recurring patterns of connected subgraphs. It has been speculated that motifs may represent modules that are used repeatedly in similar biological processes, just as transistors are reused in larger electronic circuits [11,12].

Motifs and their statistical significance were determined using the *mfinder *package [11,12] which randomizes the edges in the true network (in this case the *S. cerevisiae *full- or sub-network) among the nodes (keeping the number of nodes and the degree of each node the same as that in the true network). The frequencies of the various 4-motifs are then determined for the randomized network. This is repeated a sufficiently large number of times to give a frequency distribution for each 4-motif pattern in the ensemble of randomized networks, from which a *Z*-score for each motif can be determined [33,34]; this is defined [44] by

Z−score=n−n¯BσB;     (13)
 MathType@MTEF@5@5@+=feaafiart1ev1aaatCvAUfKttLearuWrP9MDH5MBPbIqV92AaeXatLxBI9gBaebbnrfifHhDYfgasaacH8akY=wiFfYdH8Gipec8Eeeu0xXdbba9frFj0=OqFfea0dXdd9vqai=hGuQ8kuc9pgc9s8qqaq=dirpe0xb9q8qiLsFr0=vr0=vr0dc8meaabaqaciaacaGaaeqabaqabeGadaaakeaacqWGAbGwcqGHsislcqqGZbWCcqqGJbWycqqGVbWBcqqGYbGCcqqGLbqzcqGH9aqpdaWcaaqaaiabd6gaUjabgkHiTiqbd6gaUzaaraWaaSbaaSqaaiabdkeacbqabaaakeaaiiGacqWFdpWCdaWgaaWcbaGaemOqaieabeaaaaGccqGG7aWocaWLjaGaaCzcamaabmaabaGaeGymaeJaeG4mamdacaGLOaGaayzkaaaaaa@4495@

here *n *is the number of times the motif is found in the true network n¯B
 MathType@MTEF@5@5@+=feaafiart1ev1aaatCvAUfKttLearuWrP9MDH5MBPbIqV92AaeXatLxBI9gBaebbnrfifHhDYfgasaacH8akY=wiFfYdH8Gipec8Eeeu0xXdbba9frFj0=OqFfea0dXdd9vqai=hGuQ8kuc9pgc9s8qqaq=dirpe0xb9q8qiLsFr0=vr0=vr0dc8meaabaqaciaacaGaaeqabaqabeGadaaakeaacuWGUbGBgaqeamaaBaaaleaacqWGcbGqaeqaaaaa@2F62@ is the average number of times it is found in the *B *replicate networks, and *σ*_*B *_is the standard deviation across the replicate networks. The fact that the *Z*-score is approximately normally distributed allows us to define *p*-values. Thus a *Z*-score of 4 already corresponds to *p *≈ 3.2 × 10^-5 ^and would suggest significant overrepresentation of the motif compared with the ensemble of randomized networks. It is therefore misleading to consider only the very highest *Z*-score as indicative of overrepresentation. Some authors [45] have argued that mere counting is sufficient to estimate the relative importance of a motif in a network. From a statistical perspective, such a notion cannot be upheld. We note, however, that the *Z*-score of a motif depends on an assumed probability model for network re-wiring, which may bias the *Z*-score.

## Authors' contributions

EdeS, JS., CW. and MPHS designed the study; EdeS, TT., PJI, IA, JS and MPHS analyzed the data; CW and MPHS performed the mathematical analysis; the manuscript was written jointly by EdeS, JS, CW and MPHS. All authors read and approved the final manuscript.

## Supplementary Material

Additional File 1 Variability in the degree distributions of subnets; Predicting the clustering coefficient of the overall network; Sampling properties of network components; Inferences from Motif-spectra Click here for file

## Figures and Tables

**Table 1:** Sampling fraction and sub-network size. In the present context, the true network has been taken to be the available PIN dataset (which contains itself interactions among 4773 out of an estimated 6000 *S. cerevisiae *proteins). The relationship between sampling fraction *p *and number of edges in the subnet is quadratic MS=p2MN
 MathType@MTEF@5@5@+=feaafiart1ev1aaatCvAUfKttLearuWrP9MDH5MBPbIqV92AaeXatLxBI9gBamrtHrhAL1wy0L2yHvtyaeHbnfgDOvwBHrxAJfwnaebbnrfifHhDYfgasaacH8akY=wiFfYdH8Gipec8Eeeu0xXdbba9frFj0=OqFfea0dXdd9vqai=hGuQ8kuc9pgc9s8qqaq=dirpe0xb9q8qiLsFr0=vr0=vr0dc8meaabaqaciaacaGaaeqabaWaaeGaeaaakeaacqWGnbqtdaWgaaWcbaacdaGae8NeXpfabeaakiabg2da9iabdchaWnaaCaaaleqabaGaeGOmaidaaOGaemyta00aaSbaaSqaaiab=1q8obqabaaaaa@4056@. The last line shows the extrapolation from the present network to the true network size assuming random sampling.

**Sampling fraction**	**Number of proteins**	**Mean number of interactions**
0.2	955	602
0.4	1907	2423
0.6	2864	5465
0.8	3819	9716
**1.0**	**4773**	**15181**
**Full network**	**≈6000**	**≈23700**

**Table 2:** Proteins with maximal degree-rank. The rank of a protein in the list of proteins ordered by degree, gene name, and number of connections of the top-ten most connected proteins in the full network are listed, followed by their corresponding mean rankings from the ensemble of 1000 20%, 40%, 60% and 80% subnetworks. Value in brackets are the number of subnets (out of 1000) in which the protein was present. The final column shows the degree in the high-confidence DIP data set; the correspondence between the degrees of a protein in both datasets appears to be poor. Overall, however, there is significant correlation between a protein's degree in the two data sets (*τ *≈ 0.53).

Rank Network	Gene	Degree Network	Avg. rank 20% subnet	Avg. rank 40% subnet	Avg. rank 60% subnet	Avg. rank 80% subnet	Degree in high confidence data
1	JSN1	283	1 (206)	1 (394)	1 (599)	1 (811)	–
2	CDC28	213	1.3 (193)	1.5 (416)	1.7 (585)	1.9 (797)	4
3	SRP1	197	1.3 (188)	1.7 (395)	2.1 (595)	2.5 (796)	11
4	NUP116	147	1.7 (182)	2.4 (383)	2.8 (591)	3.4 (809)	2
5	ATP14	125	2.1 (176)	2.9 (386)	3.7 (603)	4.4 (796)	–
6	SUA7	115	2.2 (193)	3.4 (414)	4.5 (616)	5.6 (806)	8
7	TEM1N	115	2.4 (183)	3.5 (402)	4.6 (597)	5.7 (791)	–
8	SRB4	109	2.6 (192)	3.8 (390)	5.2 (580)	6.7 (799)	4
9	BZZ1	107	2.6 (195)	3 (401)	5.3 (593)	6.9 (815)	1
10	VMA6	95	3.7 (193)	4.6 (414)	6.6 (582)	8.6 (788)	2
