# Supplementary material for: The effects of incomplete protein interaction data on structural and evolutionary inferences
Source: BMC Biol. 2006 Nov 3;4:39. doi: 10.1186/1741-7007-4-39 (PMC1665463; doi:10.1186/1741-7007-4-39)
Supplement: Additional File 1 — Variability in the degree distributions of subnets; Predicting the clustering coefficient of the overall network; Sampling properties of network components; Inferences from Motif-spectra [file 1741-7007-4-39-S1.pdf]

Supplementary Material

Variability in the degree distributions of subnets

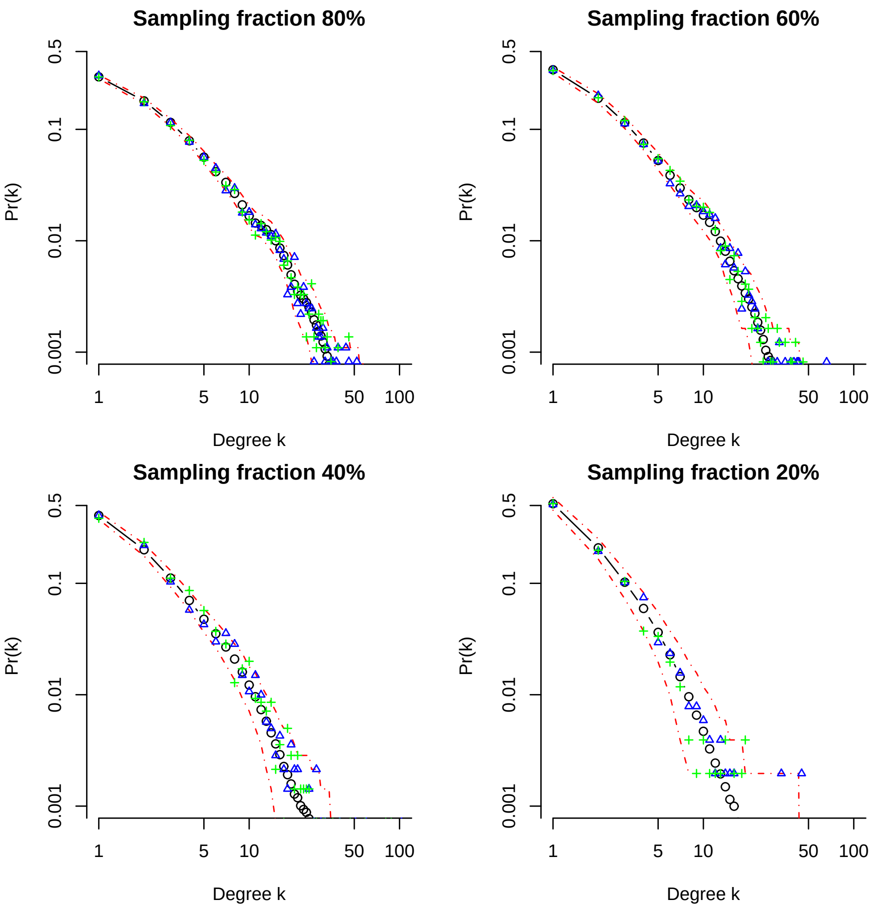

Figure 1: Average degree distributions (black circles) and empirical 95% confidence intervlas (dashed red lines) obtained from 1000 random subnets of the true *S.cerevisiae*protein interaction network. Also shown are the degree distributions of two random subnets.

In figure 1 we show the average degree distributions (black open circles), the 97.5 and 2.5 percentiles (red dashed lines) and the actual degree distributions of two random subnets. We find that the average (also shown in part A of figure 1) does describe the degree distributions well over a broad range of degrees, especially (and unsurprisingly) for larger.sampling fractions. The 95% confidence interval always broadens at higher degrees, reflecting the broad tailed (though not scale-free [5]) nature of the degree distribution. In particular small values of the sampling fraction, the CIs indicate considerable variability in the tail of the degree distributions.

Predicting the clustering coefficient of the overall network

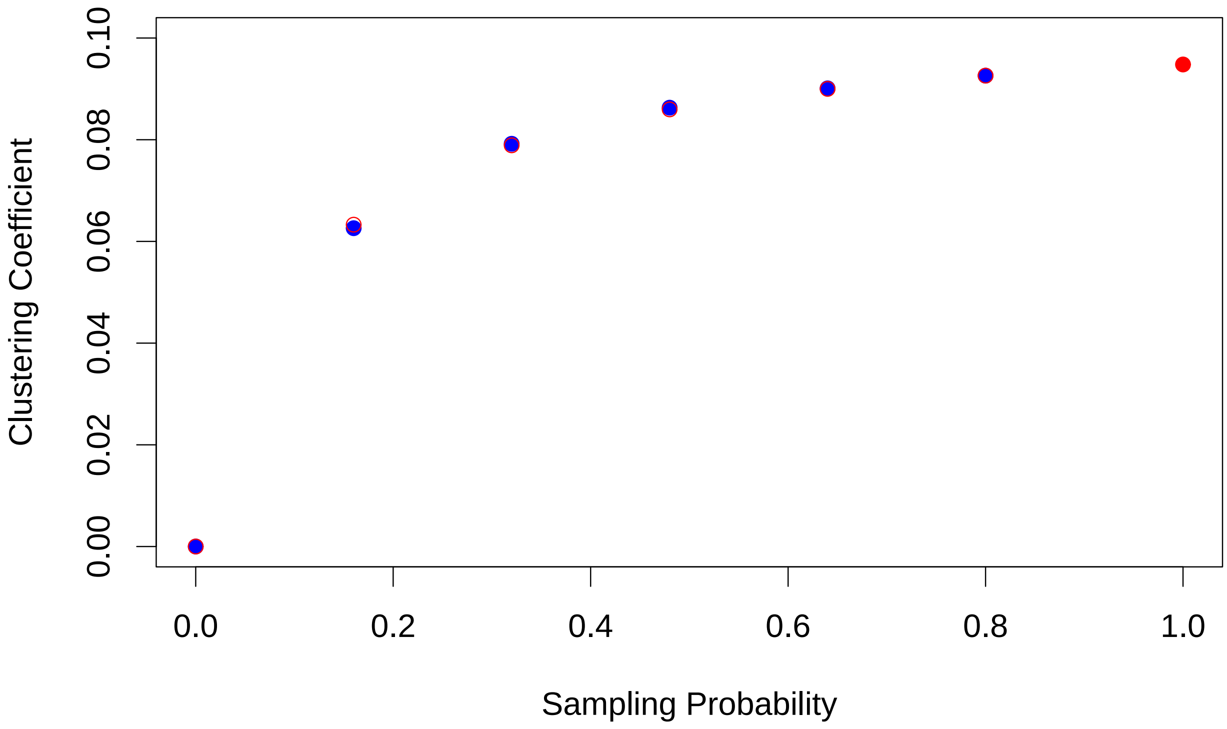

Figure 2: Observed average clustering coefficients (blue) and estimated clustering coefficients $\hat{C}_p$ (red) for different sampling fractions p. Here the full network corresponds to $p = 1.0$; thus the full dataset corresponds to the $p = 4773/6000 \approx 0.8$ in the figure. The final point is estimated from Eqn. (2).

In uncorrelated networks it is possible to express many quantities of a network in terms of the moments of the degree distribution (see box in manuscript) and for subnets of such networks we can use Eqns. (1) and (2) in the manuscript to write down approximate expressions for the clustering coefficients of the subnets etc.. Here we always assume that the network (and the subnetwork) are sufficiently large and uncorrelated.

For the (approximate) clustering coefficient[3, 2] we obtain

$$C_{\mathcal{S}} = \frac{\langle k \rangle_{\mathcal{S}}}{N_{\mathcal{S}}} \left(\frac{\langle k^2 \rangle_{\mathcal{S}} - \langle k \rangle_{\mathcal{S}}}{\langle k \rangle_{\mathcal{S}}^2} \right)^2 = \frac{p \langle k \rangle_{\mathcal{N}}}{pN} \left(\frac{p^2 \langle k^2 \rangle_{\mathcal{N}} + p(1-p)\langle k \rangle_{\mathcal{N}} - p\langle k \rangle_{\mathcal{N}}}{p^2 \langle k \rangle_{\mathcal{N}}^2} \right) = C_{\mathcal{N}}, \quad (1)$$

i.e. in an uncorrelated uniform network the clustering coefficient of a random subnet will be the same as that of the overall network.

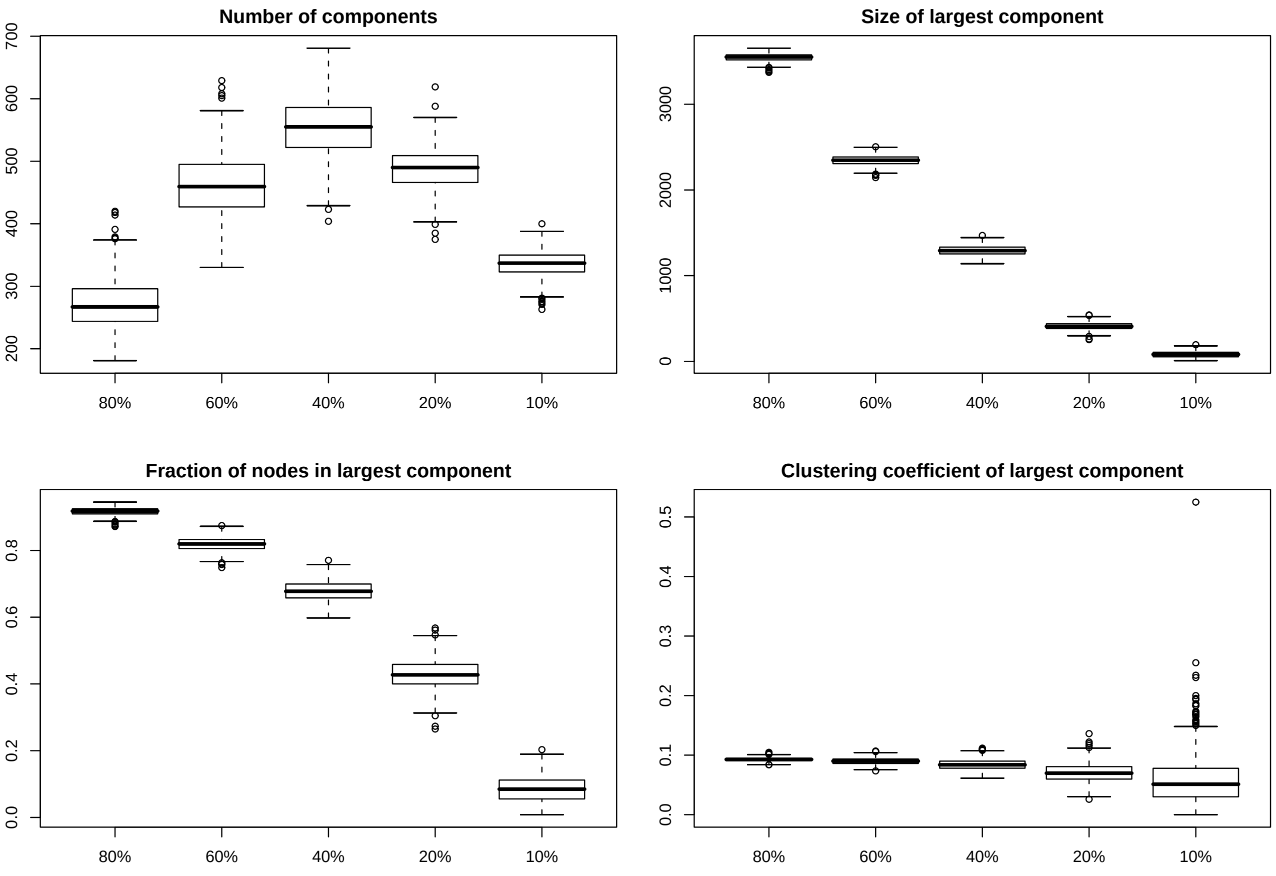

Figure 3: Analysis of component numbers and properties of the giant connected component.

In reality, however, (see figure 1 of the manuscript) we observe that the clustering coefficient in the subnets is significantly smaller than that of the true network. The decrease in C with decreasing size in the *S.cerevisiae*datasets is much faster than the decrease in C in ensembles of classical random graphs of the same size.

Interestingly, we found a very simple functional dependence between the clustering coefficient as a function of sampling fraction, p,

$$\hat{C}_p = \frac{p}{\gamma \times p + 1} \tag{2}$$

where γ can be determined from the fit to the observed data and we obtain $\gamma \approx 9.549$. This allows us to estimate the clustering coefficient of the true network (*i.e.* the yeast interactome defined by the experimentally accessible interactions among the approximately 6000 proteins) of $\hat{C}_{1.0} = 0.095$. The fit of this simple, single parameter function to the observed data is very good (see figure 2)

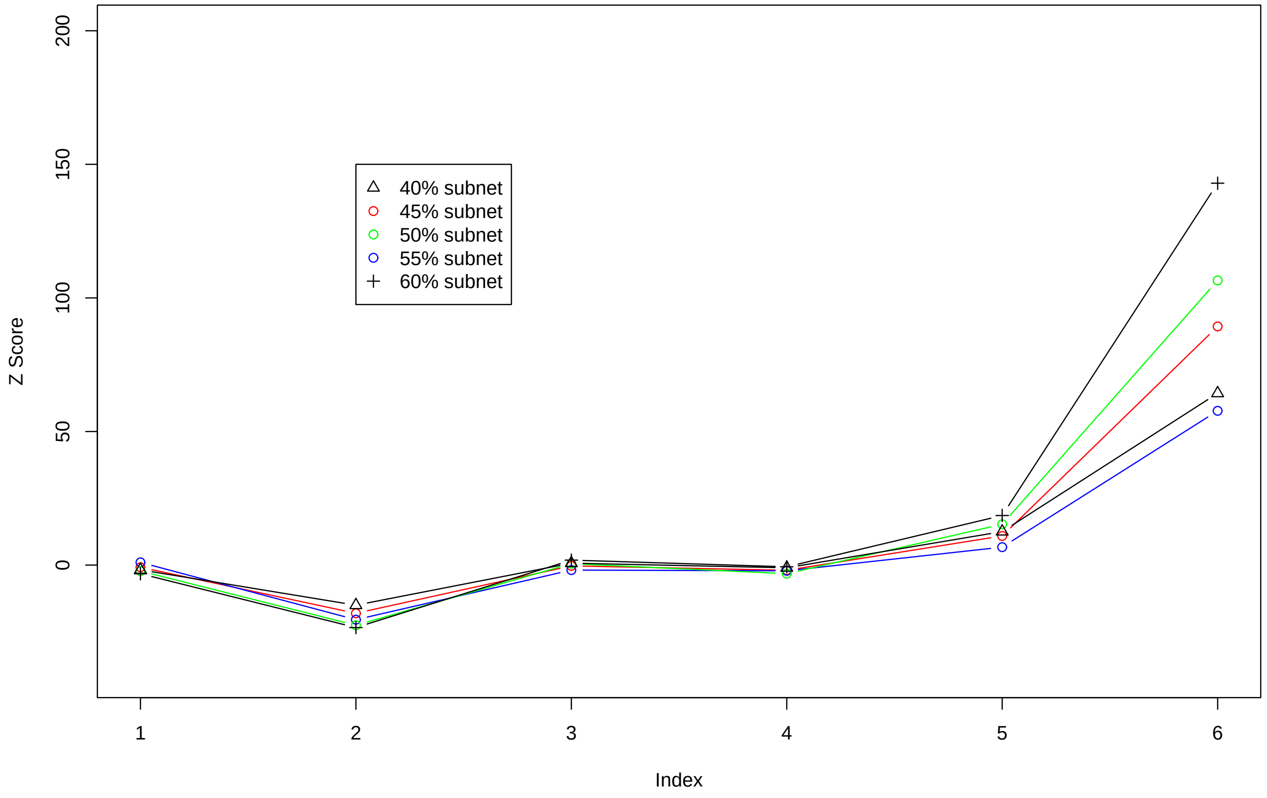

Figure 4: Median motif spectra for sampling fractions $p = 0.4, 0.45, 0.5, 0.55$ and 0.6.

Sampling properties of network components

In figure 3 we show how connected components, in particular the giant connected component, are affected by random sampling of nodes. For $p = 10\%$ we are approaching the phase transition where the giant component vanishes. This is clearly seen in the figure. Notice that the number of components increases first with decreasing p before decreasing. This occurs when many components contain only one node and the probability of not sampling such single nodes becomes larger than the probability of further breaking up other larger components.

Many important network properties will be influenced by the loss of the GCC; most notably this applies to the non-local phenomena such as average pathlengths, network diameter, betweenness and closeness [1, 2, 4]. Note however, that for many networks with a broad-tailed degree distribution, Eqn. (6) in the manuscript can be approximated by

$$\frac{\langle k \rangle_{\mathcal{N}}}{\langle k^2 \rangle_{\mathcal{N}} - \langle k \rangle_{\mathcal{N}}} = \frac{\langle k \rangle_{\mathcal{N}}}{\langle k^2 \rangle_{\mathcal{N}}} \tag{3}$$

which will tend to be small (≈ 0.04 in the context of the present Yeast data) such that the

GCC should persist for most present PIN datasets even if they were generated by random sampling of nodes. Deviation from the random sampling scheme will of course alter results considerably. In any realistic experimental setup we would expect to see a GCC.

Inferences from Motif-spectra

In the manuscript (figures 1C,D and 2C) we have have seen that motifs, especially the most connected 4-motif are subject to considerable variation in different instances of subnets of the same size. Perhaps more worryingly we found that the Z-score distributions for different sampling probabilities p overlap. This is also confirmed in figure 4 where we show median Z-scores obtained from 20 replicates for five different sampling fractions $p = 0.4, 0.45, 0.5, 0.55, 0.6$. The lines connecting the Z-score spectra/profiles cross several times and the rank order of z-scores follows no uniform trend.

References

[1] E. de Silva and M.P.H. Stumpf. Complex networks and simple models in biology. *J.Roy.Soc. Interface*, 2005.

[2] S.N. Dorogovtsev and J.F.F. Mendes. *Evolution of Networks*. Oxford University Press, 2003.

[3] H. Ebel, L.I. Mielsch, and S. Bornholdt. Scale-free topology of e-mail networks. *Phys.Rev.E*, 66(035103), 2002.

[4] T.S. Evans. Complex networks. *Contemporary Physics*, 45(6):455–474, 2004.

[5] M.P.H. Stumpf, P.J. Ingram, I. Nouvel, and C. Wiuf. Statistical model selection methods applied to biological networks. *Trans.Comput.Systems Biol.*, 3:65–72, 2005.
